# Quasispecies composition and evolution of a typical Zika virus clinical isolate from Suriname

**DOI:** 10.1038/s41598-017-02652-w

**Published:** 2017-05-24

**Authors:** Sander van Boheemen, Ali Tas, S. Yahya Anvar, Rebecca van Grootveld, Irina C. Albulescu, Martijn P. Bauer, Mariet C. Feltkamp, Peter J. Bredenbeek, Martijn J. van Hemert

**Affiliations:** 1 Department of Medical Microbiology, Leiden University Medical Center, Leiden, The Netherlands; 2 Department of Human Genetics, Leiden University Medical Center, Leiden, The Netherlands; 3 Department of Infectious Diseases, Leiden University Medical Center, Leiden, The Netherlands

## Abstract

The arthropod-borne Zika virus (ZIKV) is currently causing a major international public health threat in the Americas. This study describes the isolation of ZIKV from the plasma of a 29-year-old female traveler that developed typical symptoms, like rash, fever and headache upon return from Suriname. The complete genome sequence including the 5′ and 3′ untranslated regions was determined and phylogenetic analysis showed the isolate clustering within the Asian lineage, close to other viruses that have recently been isolated in the Americas. In addition, the viral quasispecies composition was analyzed by single molecule real time sequencing, which suggested a mutation frequency of 1.4 × 10^−4^ for this ZIKV isolate. Continued passaging of the virus in cell culture led to the selection of variants with mutations in NS1 and the E protein. The latter might influence virus binding to cell surface heparan sulfate.

## Introduction

Zika virus (ZIKV) is a mosquito-borne member of the *Flaviviridae* family. It is primarily transmitted through mosquitoes of the *Aedes* genus^1^. First isolated in 1947^2^ and having caused only sporadic or unnoticed infections for about 60 years, the virus has received little attention until it emerged on several islands in the Pacific ocean in 2007^3^. Since 2015, ZIKV is causing a major outbreak, affecting millions, in Brazil and other countries in South and Central America^4, 5^. Many infected travelers, often asymptomatic, have returned to other parts of the world and between June and August 2016 the first local mosquito-transmitted ZIKV infections occurred in the USA^6^.

ZIKV has a positive-stranded RNA genome that is capped, lacks a poly(A) tail and has a typical flavivirus genome organization. It contains a single open reading frame that encodes the 3 structural and 7 nonstructural proteins in the form of a polyprotein. ZIKV strains can be classified in two separate lineages, the African and Asian lineages. Both lineages have emerged from East Africa around the end of the 19^th^ century^7^. The viruses that are spreading in the Americas are very similar to the African genotypes that have been isolated decades ago, but based on phylogenetic studies they were shown to be most closely related to the Asian lineage strain that circulated in French Polynesia in 2013^5, 8, 9^.

ZIKV causes asymptomatic infections in about 80% of the cases and mild dengue-like symptoms in the majority of symptomatic patients, including fever, myalgia, headache and a macopapular rash. However, in pregnant women ZIKV infections can result in serious damage to the fetus, causing neurologic disorders and neonatal malformations, like microcephaly^10^. In rare cases, ZIKV-infected adults can develop a paralytic neurological complication known as the Guillain-Barré syndrome^11, 12^. Besides transmission by mosquitos, ZIKV can also utilize a sexual mode of transmission^13–15^. Blood or urine is generally used to diagnose ZIKV infections, but viral RNA has been found in other body fluids and tissues as well. To date, no registered vaccine or antiviral therapy is available to prevent or treat ZIKV infections.

The RNA-dependent RNA polymerases of RNA viruses lack proofreading ability, leading to high mutation rates in comparison to cellular organisms^16^. These high mutation rates result in a high genetic variation within the virus population, the so-called quasispecies, a set of genetically related viruses closely distributed around a consensus sequence^17, 18^. Next-generation sequencing (NGS) has enabled the analysis of the population of viral quasispecies with a much higher resolution than conventional sequencing. The characterization of the ZIKV quasispecies composition and (intrahost) evolutionary dynamics in relation to adaptation, virulence, and immune escape is important to better understand ZIKV infection and pathogenesis, and to aid development of vaccines and therapeutics.

In the present study, we report and analyze the complete genome sequence and quasispecies distribution of a ZIKV strain from Suriname isolated from the plasma of a 29-year-old female traveler who returned to the Netherlands and exhibited typical symptoms of ZIKV infection.

## Results

### Case description

**Figure 1:**
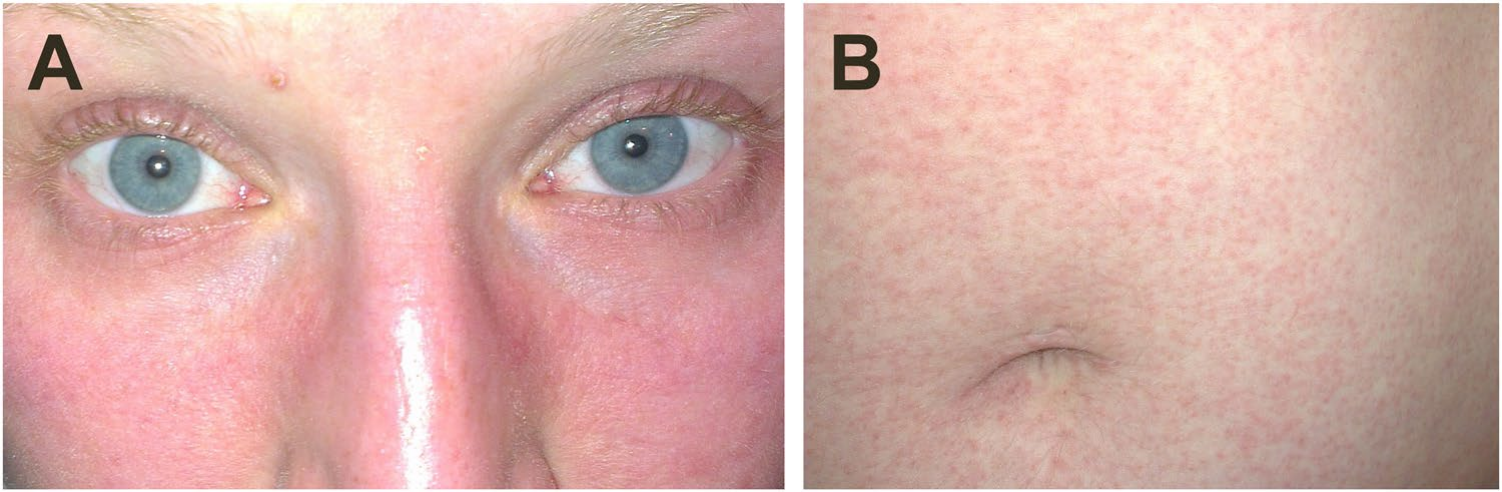
Photographs of (**A**) erythema in the face and slightly engorged blood vessels in the eyes, and (**B**) non-itching maculopapular rash on the abdomen of the patient from which ZIKV SL1602 was isolated.

A 29-year-old female medical microbiologist in training developed a low grade fever, general malaise and a retro-orbital headache related to eye movements three days after returning from Suriname. Three days later, the patient noticed a redness in her face, a non-itching maculopapular rash on her trunk and extremities, and slight conjunctival injection, which she recorded on photo (Fig. 1). On the same day the patient developed stiffness and edematous swelling of wrists, knees and ankles. Throughout the course of disease she had contact lens intolerance. The symptoms did not lead to lost working days and diminished after 7 days. On the fifth day of her illness she visited our outpatient clinic where urine and blood was collected for diagnosis and characterization of an arboviral infection. Urine and plasma tested positive for ZIKV RNA by an in-house qRT-PCR (manuscript in preparation), and the plasma also tested positive in virus culture. No infectious virus could be recovered from the urine. The patient had been bitten by mosquitos in Suriname where she had visited Paramaribo, the Brokopondo Lake and the Commewijne district.

### Isolation and sequencing of ZIKV strain SL1602

Plasma of the patient (12 μL) was added to a semi-confluent culture of Vero E6 cells. After 4 days the cell culture medium, which tested positive for ZIKV-RNA in qRT-PCR, was harvested (P1 stock) and 1 ml was used to infect a fresh batch of Vero E6 cells for subsequent passaging. The P1 stock had a ZIKV titer of 9.0 × 10^2^ pfu/ml, while the P2 stock had a titer of 8.2 × 10^5^ pfu/ml, as determined by plaque assay on Vero cells. Attempts to isolate infectious ZIKV from urine were unsuccessful.

Viral RNA was isolated from the P2 virus stock (cell culture supernatant) and 5 partially overlapping amplicons were generated. A near complete genome sequence was first obtained by Sanger sequencing. The sequences of the 5′ and 3′ termini, which are frequently missing or incorrect in other ZIKV sequences in GenBank, were determined by rapid amplification of cDNA ends (5′- and 3′-RACE). To obtain the consensus complete genome sequence of ZIKV SL1602, we combined Sanger sequencing, 5′- and 3′-RACE data, and Pacific Biosciences RSII single molecule real time (SMRT) sequencing. The complete genome of this isolate consists of 10,807 nucleotides with an overall G/C content of 51.2%.

The quasispecies distribution was determined by subjecting the 5 overlapping amplicons to SMRT sequencing. The data from the SMRT deep-sequencing run yielded a total of 31,421 high-quality single-molecule sequencing reads, with ZIKV genome coverage ranging from 427 to 5,831 reads at single nucleotide positions, with an average of 2,300 reads (Figure S1). The minimum number of passes for each single molecule read was 5 and the maximum number of passes was 183, with a median of 12. This led to the high minimum read quality of 0.99 and an average read quality of 0.998.

### Phylogenetic relationships between ZIKV SL1602 and other isolates

**Figure 2:**
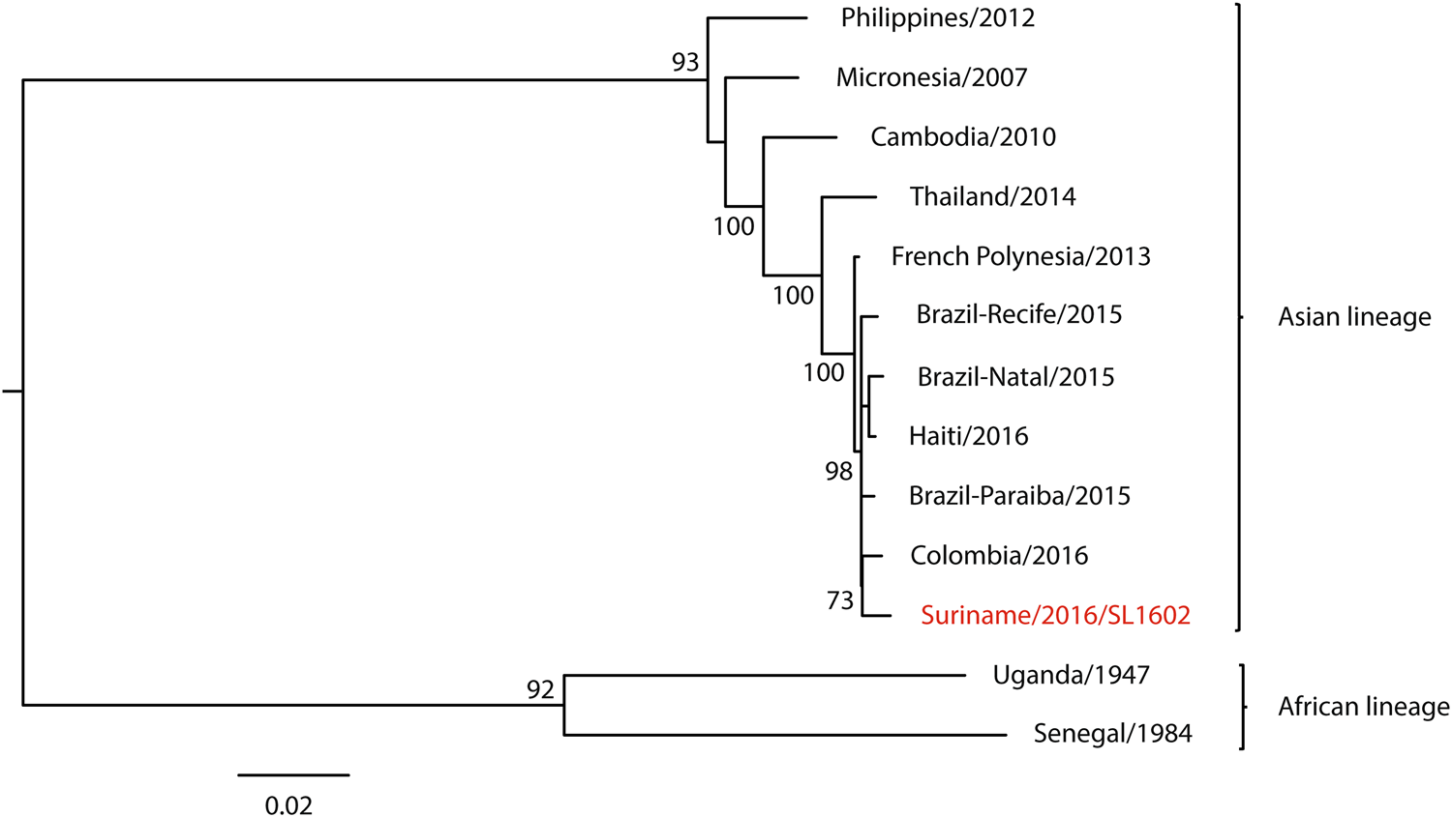
Phylogenetic tree for ZIKV SL1602 and 12 selected ZIKV isolates that represent the recognized diversity. A maximum likelihood phylogenetic tree inferred from the nucleotide sequences of full-length ZIKV genomes is shown. The country of isolation and GenBank accession numbers of the isolates that were used are: Uganda MR 766 (NC012532), Brazil-Natal (KU527068), Thailand (KU681081), Philippines (KU681082), Brazil-Paraiba (KX280026), Colombia (KX247646), Brazil-Recife (KX197192), Haiti (KX051563), Senegal (KU955591), Cambodia (KU955593), Federated States of Micronesia (EU545988) and French Polynesia (KJ776791.2). Spondweni virus (NC029055, not shown) was used as the outgroup. Bootstrap values above 70 are shown. The scale bar represents the number of nucleotide substitutions per site.

To understand the evolutionary relationship of ZIKV SL1602, a maximum likelihood phylogenetic tree was inferred using ZIKV complete genome nucleotide sequences (Fig. 2). The tree was produced for a spatiotemporal representative set of ZIKV strains for which complete genome sequences were available in GenBank. ZIKV SL1602 clusters within the Asian lineage, relatively close to viruses that were or are still circulating in French Polynesia, Brazil, Haiti, and Colombia. This suggests that the 2015/2016 ZIKV isolates in American and Asian countries form a new American clade within the Asian lineage. Although the phylogenetic distance between the isolates of the Asian lineage is very small, the Columbia/2016 isolate is the closest relative of ZIKV SL1602 with only 44 nucleotides difference thereby having a 99.5% sequence identity.

### Quasispecies variation

SMRT sequencing reads were aligned to the consensus sequence and for each nucleotide position the count for each of the four bases was determined. A total of 24,815,877 nucleotides was sequenced and 3,375 single-nucleotide variants (SNV) were counted. This suggests that ZIKV has a mutation frequency of 1.4 × 10^−4^, which falls within the range that has been reported for other RNA viruses^19^.

**Figure 3:**
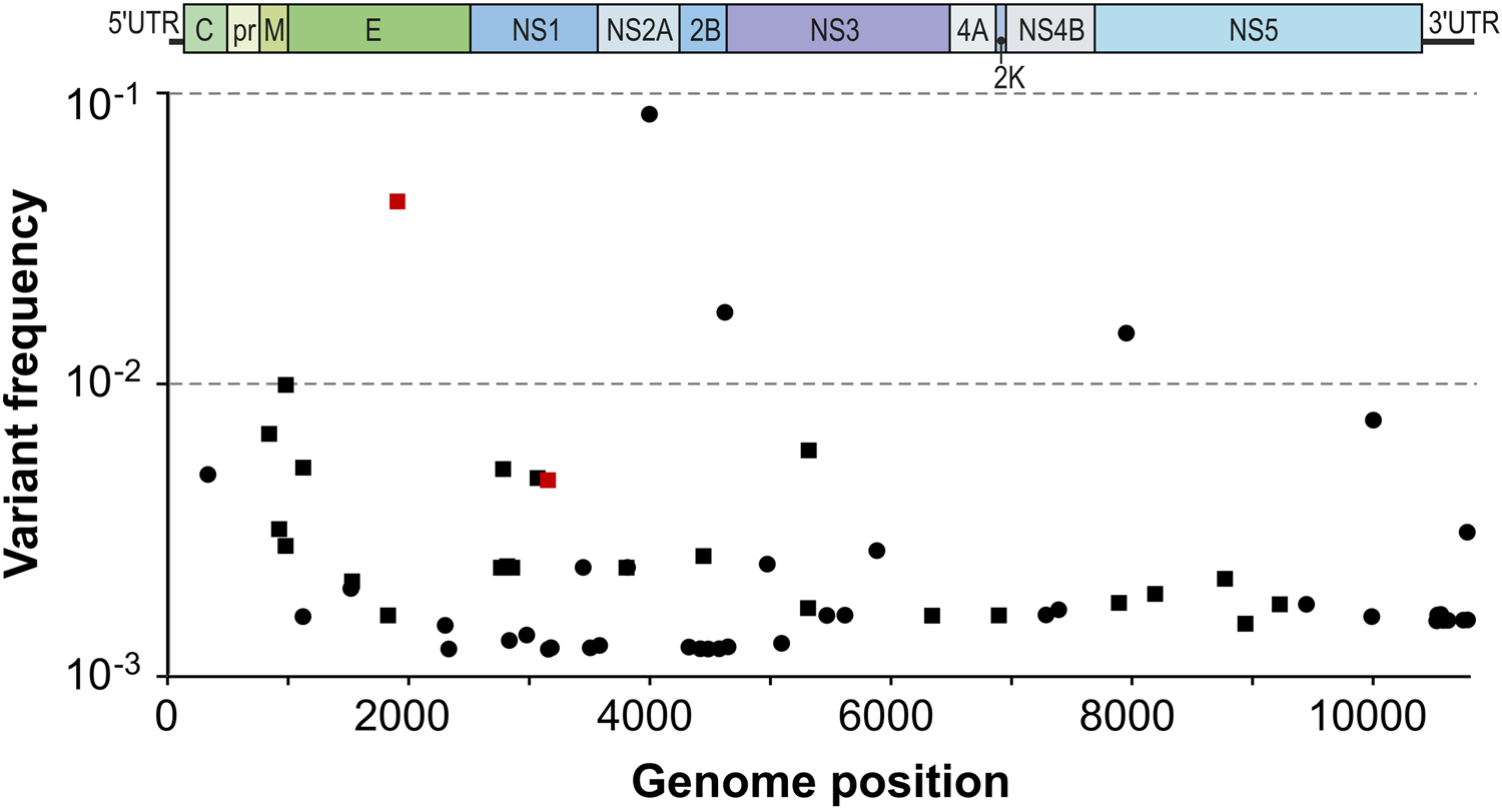
Quasispecies population of ZIKV SL1602. Synonymous substitutions are indicated by dots and nonsynonymous substitutions by squares. Red squares indicate SNVs that became dominant (fixed) when the virus was passaged 9 more times on Vero cells.

Seventy-one SNVs were mapped using coverage-corrected cut-off values (Fig. 3). Their frequencies ranged from 0.0012 to 0.0835. Out of the 71 SNVs, 27 (38%) were non-synonymous (Table 1), and 44 (62%) were synonymous mutations (Supplemental Table 2).

**Table 1:** Non-synonymous SNVs in the ZIKV SL1602 quasispecies population.

genome position	reference nt	mutation	frequency	amino acid change	protein
834	C	U	0.0067	H243Y	M
917	C	A	0.0031	S270R	M
970	C	U	0.0027	A288V	M
972	U	C	0.0098	Y289H	M
1116	A	G	0.0051	T337V	E
1522	G	U	0.0021	G472V	E
1820	G	A/C	0.0008/0.0008	K571K/N	E
1898	G	U	0.0419	L597F	E
2758	U	C	0.0023	V884A	NS1
2774	C	A	0.0051	N889K	NS1
2781	U	C	0.0023	W892R	NS1
2808	C	A/U	0.0011/0.0011	P901T/S	NS1
2851	C	U	0.0023	S915L	NS1
3061	A	G	0.0047	K985R	NS1
3147	A	G	0.0046	M1014V	NS1
3798	G	C	0.0023	V1231L	NS2A
4437	C	U	0.0025	P1444S	NS2B
5306	A	U	0.0017	E1733D	NS3
5312	G	U	0.0058	E1735D	NS3
6336	A	G	0.0016	S2077G	NS3
6889	U	C/G	0.0005/0.0010	V2261A/G	2 K
7885	C	A	0.0018	P2593H	NS5
8185	A	G	0.0019	E2693G	NS5
8765	C	A/U	0.0007/0.0014	D2886E/D	NS5
8934	G	A/U	0.0012/0.0002	A2943T/S	NS5
8935	C	U	0.0015	A2943V	NS5
9219	U	C/G	0.0002/0.0014	Y3038H/D	NS5

Four variants in the quasispecies population were found to be present at a level of over 1% of the total population. One of these variants contained a mutation in the E protein (4.1%), and the other three contained mutations in the nonstructural proteins NS2A (8.2%), NS2B (1.6%), and NS5 (1.4%) (Fig. 3).

The passage 2 (P2) ZIKV isolate that was sequenced was passaged 9 more times in Vero cells to obtain a P11 stock, which was analyzed by Sanger sequencing. The consensus sequence of the P11 virus differed at two nucleotide positions from the P2 virus. It has the G1898U, mutation, which results in a L307F substitution in the E protein, and the A3147G mutation, which leads to a M220V substitution in the NS1 protein. For both positions, SNVs were already observed in the P2 quasispecies, with a frequency of 0.0419 and 0.0046, respectively (Table 1). Sequencing of virus from earlier passages (P5 and P8 virus) revealed that both changes were fixed between passage 9 and 11. These changes likely reflect an adaptation to Vero cells.

## Discussion

Here, we report the isolation and analysis of a ZIKV strain from a traveler returning from Suriname with typical symptoms of Zika. The complete genome was sequenced, a phylogenetic analysis was performed and single nucleotide variations were analyzed to characterize the quasispecies composition of this clinical isolate that caused commonly observed mild symptoms.

The analysis of the quasispecies distribution of ZIKV SL1602 was done on the Pacific Biosciences RSII, which allows the rapid sequencing of relatively large DNA molecules. Only five PCR fragments were used to sequence the complete genome, without additional library preparation steps (e.g. fragmentation). Although SMRT sequencing is prone to random high sequencing errors, every single amplicon molecule was sequenced a minimum of 5 times (Figure S2), leading to a very high accuracy of base calling (Figure S3).

Our phylogenetic analysis showed that ZIKV SL1602 clusters within the Asian lineage, close to the viruses that are currently circulating in the Americas (Fig. 1), in accordance with other studies^8, 20–22^. All of the South American isolates share a common ancestor with the French Polynesia isolate, suggesting that the currently spreading South American clade is imported from French Polynesia.

ZIKV SL1602 has a typical flavivirus genome organization with one large open reading frame that encodes the polyprotein C-prM-E-NS1-NS2A-NS2B-NS3-NS4A-NS4B-NS5 that will be processed into 3 structural and 7 nonstructural proteins. The sequence reported here includes the complete 5′ and 3′ untranslated regions, which are often lacking from other sequences that have been deposited in public databases.

RNA viruses have high mutation rates and evolve rapidly due to the lack of proofreading of the RNA-dependent-RNA-polymerase. Our NGS analysis shows that the mutation frequency of ZIKV SL1602 is 1.4 × 10^−4^, which is within the range commonly observed for RNA viruses (10^−4^ to 10^−6^)^19^. We did not observe a preferential positive selection for mutations in nonstructural proteins, as was described before^23^.

Repeated passaging of ZIKV SL1602 in Vero cells led to selection of a variant with an L307F substitution in the E protein and a M220V substitution in NS1. The L307F variant was already present in the P2 quasispecies with a frequency of 0.0419. Based on its structure, the flavivirus E protein can be divided into domains I, II and III^24^ of which domain III is believed to play a major role in antibody binding, pathogenesis and attachment to host cells. The L307F mutation is located in domain III of the ZIKV E protein. It might therefore influence cell attachment and infectivity, since domain III was shown to contribute to the tropism, infectivity, pathogenesis and cell attachment of other flaviviruses, possibly involving cell surface heparan sulfate binding^25–27^. The M220V mutation in NS1 that was fixed during repeated passaging in Vero cells is a conservative replacement. However, considering the role of NS1 in DENV particle biogenesis^28^, it cannot be excluded that it can contribute to increased replication or other selective advantages in cell culture.

By analyzing the complete genome sequence of a South American (Surinamese) ZIKV isolate, our study shows the quasispecies composition and minority variants of ZIKV early after isolation from a patient, and upon repeated passaging in cell culture. Insights into the evolutionary dynamics of ZIKV can aid in the development of improved diagnostics, surveillance, development of vaccines and therapeutics, and thereby in controlling further spread of ZIKV.

## Methods

### Cell culture, virus isolation and titration

Vero cells (ATCC CCL-81) and Vero E6 cells (ATCC CRL-1586) were cultured in Dulbecco’s modified Eagle’s medium (DMEM), supplemented with 8% fetal calf serum (FCS), 2 mM L-glutamine, 100 IU/ml of penicillin and 100 μg/ml of streptomycin at 37 °C in 5% CO_2_. After infection, cells were maintained in Eagle’s Minimum Essential Medium (EMEM) with 25 mM HEPES supplemented with 2% FCS, L-glutamine, and antibiotics. All experiments with infectious ZIKV were performed in the LUMC BSL-3 facility. Written informed consent was obtained from the patient to publish images that show the symptoms of infection and to use clinical samples taken for diagnosis for scientific research, including the isolation of virus. A semi-confluent monolayer of Vero E6 cells in a T-25 flask was incubated with 12 µl of plasma in a total volume of 5 ml medium. After 4 days, 1 ml of supernatant was transferred to a new T-25 flask with Vero E6 cells already containing 5 ml of medium and 8 days later the medium was harvested and stored as a P2 stock. Viral titers were determined by plaque assay on confluent monolayers of Vero cells in six-well clusters, which were infected with 10-fold serial dilutions (in duplicate) of the samples in a total volume of 0.5 ml. After a 2-h incubation at 37 °C, the inoculum was replaced with 2 ml of DMEM containing 1.2% Avicel RC-581 (FMC BioPolymer), 2% FCS, 25 mM HEPES, and antibiotics. After a 5-day incubation, cells were fixed with 3.7% formaldehyde in PBS and plaques were visualized by crystal violet staining. ZIKV RNA was detected by an internally controlled multiplex TaqMan qRT-PCR (manuscript in preparation).

### ZIKV RNA isolation and generation of cDNA

ZIKV-specific primers to generate 5 overlapping amplicons were designed based on an alignment of the reference sequence ZIKV strain MR766 and other complete genomes present in GenBank in February 2016 (Table S1).

ZIKV RNA was isolated from infected cell culture medium using the Qiamp Viral mini kit (Qiagen) and cDNA was generated by reverse transcription (5 separate reactions) with Superscript III polymerase (Thermo Fisher Scientific) and primers A1R, A2R, A3R, A4R and A5R (Table S1) according to the supplier’s instructions. Subsequently, 5 µl cDNA was used in 50 µl PCR reactions with Q5 high-fidelity DNA Polymerase (NEB) with GC enhancer to generate 5 amplicons with sizes of 2.4, 3.3, 3.1, 2.5 and 0.54 kb in 5′ to 3′ order, using primer combinations A1F & A1R, A2F & A2R, A3F & A3R, A4F & A4R and A5F & A5R. An initial step of 30 s at 98 °C was followed by 35 cycles consisting of a 10 s denaturation step at 98 °C, a 30-s annealing step at 64 °C and a 140 s extension at 72 °C for amplicons 1–4, while a 66 °C annealing temperature and 40 s extension time was used to generate amplicon 5. After cleanup with the ISOLATE II PCR Kit (Bioline), DNA was quantified using the Qubit Fluorometer and dsDNA BR Assay (Thermo Fisher Scientific). It was analyzed on agarose gels and the quality was checked with a Bioanalyzer DNA 12000 Kit (Agilent).

### Full genome sequencing

ZIKV cDNA amplicons were subjected to Sanger sequencing with overlapping reads that covered the amplicons on both strands (primer sequences available upon request). For sequencing of the 5′ and 3′ termini of the genome, isolated ZIKV RNA was first polyadenylated using yeast poly(A) polymerase (Affymetrix), followed by rapid amplification of cDNA ends (RACE). RACE was done with the 2nd Generation 5′/3′ RACE kit (Roche) using primers SP1, SP2, SP3, SP5 (Table S1) and the primers included in the kit, according to the manufacturer’s instructions. The 5′ and 3′ amplicons were sequenced using primers 5RACE-seq1 and 3RACE-seq, respectively (Table S1).

For NGS, the 5 amplicons were gel-purified, pooled in equimolar concentrations and library preparation was performed according to the Pacific Biosciences Amplicon Template Preparation protocol. Sequencing was performed on a single SMRT cell using a 6-hour runtime and the C4-P6 chemistry on the RSII platform. Reads-of-inserts with a minimum of 5 full passes and minimum predicted accuracy of 99% were subsequently mapped to the ZIKV SL1602 reference sequence using SMRT Analysis software version 2.3. For each amplicon, primer sequences were removed and nucleotide counts were generated for each genomic position.

### Mutation frequency

All reads derived from SMRT sequencing were aligned to the ZIKV SL1602 consensus sequence. From this alignment, a table was created that contained for each genome position the counts for each of the four nucleotides. The ‘total number of mutations’ was the sum of all counts of the nucleotides that differed from the consensus sequence. The ZIKV mutation frequency was calculated by dividing the total number of mutations by the total number of nucleotides sequenced.

### Phylogenetic reconstruction

Nucleotide sequences were aligned using the ClustalW software running within the BioEdit (version 7.2.5) program^29^. This resulted in a 10,302 nt alignment. Maximum likelihood phylogenetic trees with 1000 bootstrap replicates were estimated under the general time-reversible model (GTR)+G+I+F using PhyML 3.0 software^30^.

### Sequence accession number

The ZIKV SL1602 consensus sequence, based on PacBio and Sanger sequencing (RACE) was submitted to GenBank under accession number KY348640.

## Electronic supplementary material

Dataset 1
