# Supplementary material for: Quasispecies composition and evolution of a typical Zika virus clinical isolate from Suriname
Source: Sci Rep. 2017 May 24;7:2368. doi: 10.1038/s41598-017-02652-w (PMC5443807; doi:10.1038/s41598-017-02652-w)
Supplement: Supplementary file 1 — Dataset 1 [file 41598_2017_2652_MOESM1_ESM.pdf]

Supplemental Material with manuscript SREP-17-06278-T

Quasispecies composition and evolution of a typical Zika virus clinical isolate from Suriname

Sander van Boheemen[1], Ali Tas[1], S. Yahya Anvar[2], Rebecca van Grootveld[1], Irina C. Albulescu[1], Martijn P. Bauer[3], Mariet C. Feltkamp[1], Peter J. Bredenbeek[1] and Martijn J. van Hemert[1*].

Affiliations:

1) Department of Medical Microbiology, Leiden University Medical Center, Leiden, The Netherlands.

2) Department of Human Genetics, Leiden University Medical Center, Leiden, The Netherlands.

3) Department of Infectious Diseases, Leiden University Medical Center, Leiden, The Netherlands.

* Corresponding author: M.J. van Hemert, Department of Medical Microbiology, Leiden University Medical Center, Albinusdreef 2, 2333 ZA Leiden. The Netherlands. Phone: +31 71 5266793. E-mail: M.J.van_Hemert@lumc.nl.

Supplemental table 1. Sequences of primers used for SMRT sequencing and determining the sequence of the 5’ and 3’ terminal regions of the genome.

**primer**	**sequence**
A1F	AGTTGTTGATCTGTGTGAATCAG
A1R	GCCAAGCACATAAGGGAAATAG
A2F	TGAGAGGTGCCAAGAGAATG
A2R	GAAGTGGGCCTCATCCATAATA
A3F	GCCTTCGATGCTGAAGAAGA
A3R	CTCCAGGGTTTCCATCATAGTG
A4F	CCGCAAAGTTCAAGAAGTGAAAG
A4R	CGTTCTCGGCCTGACTATAG
A5F	AACACAGTCAACATGGTGCG
A5R	GAAACCATGGATTTCCCCACACCGGCCGCC
SP1	GTGATGGCAGGTTCCGTACA
SP2	TCGTGTTGCACCAACAATCG
SP3	TCCCCAGCATCGTTTCTGTC
SP5	CAAGCCTATAGTCAGGCCGAG
5RACE-seq1	AGGCCAACAATTCCGACACT
3RACE-seq	ATGGGAAAAGAAGGTGGCGA

Supplemental table 2. Synonymous SNVs in the ZIKV SL1602 quasispecies population.

genome position	reference nt	mutation	frequency
326	G	A	0.0048
1115	U	A/C	0.0016
1511	C	U	0.0020
2294	C	U	0.0015
2324	U	C	0.0012
2826	C	C	0.0013
2970	G	G	0.0014
3151	A	A	0.0012
3172	C	C	0.0012
3173	C	C	0.0012
3174	C	C	0.0012
3440	A	G	0.0023
3498	C	C	0.0012
3576	C	C	0.0013
3806	A	G	0.0023
3989	U	C	0.0835
4316	C	C	0.0012
4412	A	A	0.0012
4477	C	C	0.0012
4568	C	C	0.0012
4616	U	C	0.0174
4641	C	C	0.0012
4967	A	G	0.0024
5085	A	A	0.0013
5462	U	C	0.0016
5612	U	C	0.0016
5876	C	U	0.0026
7280	C	U	0.0016
7385	U	C	0.0017
7946	C	U	0.0148
9440	U	C	0.0017
9983	G	A	0.0016
9995	G	A	0.0074
10521	C	A	0.0015
10529	C	U	0.0016
10550	A	G	0.0016
10551	A	G	0.0016
10556	C	A	0.0016
10571	G	A	0.0015
10589	A	U	0.0015
10614	A	G	0.0015
10743	C	U	0.0015
10774	U	C	0.0031
10777	C	U	0.0015

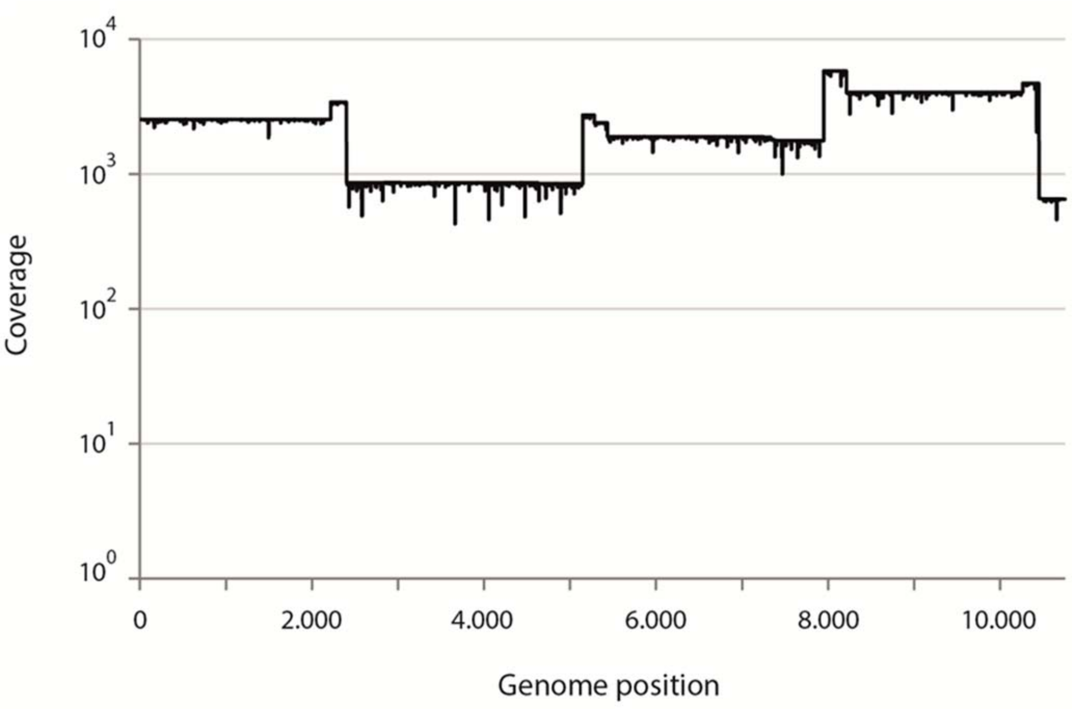

Supplemental figure 1. Coverage plot of the ZIKV SL1602 genome determined by SMRT sequencing.

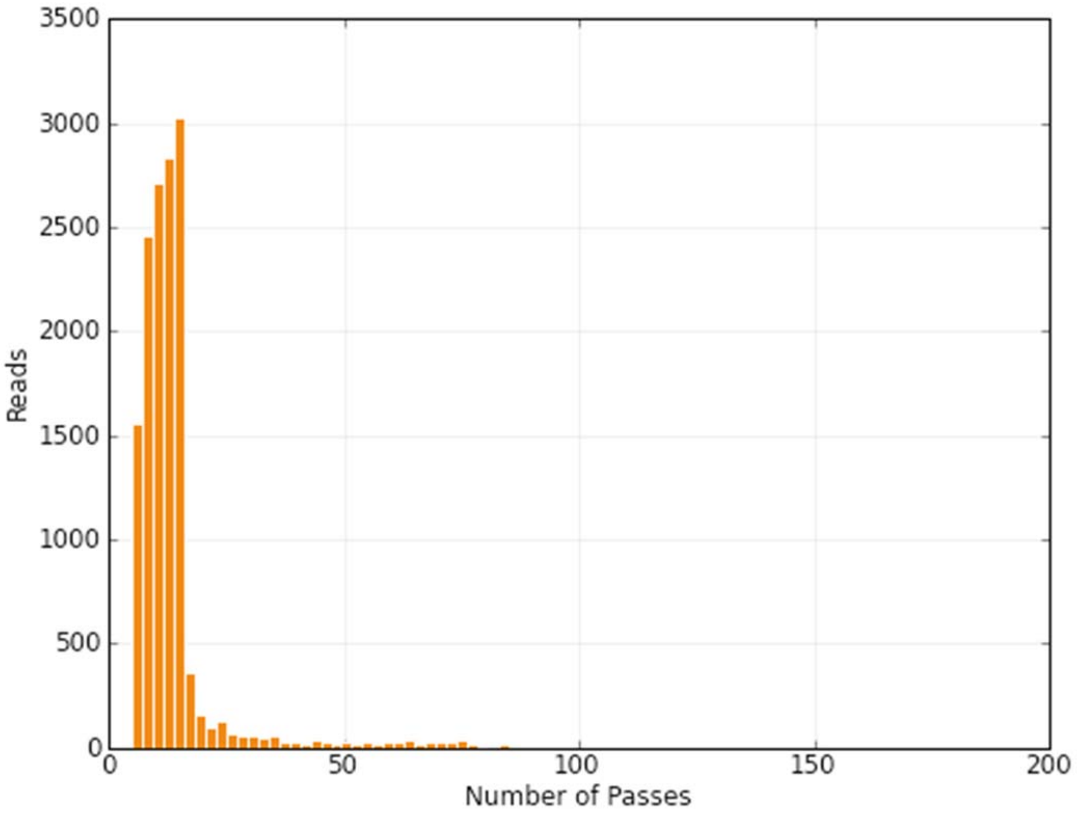

Supplemental figure 2. Multi-pass single molecule sequencing of generated amplicons of ZIKV SL1602. Histogram illustrates the number of times each molecule is sequenced by Pacific Biosciences RSII platform to generate the circular-consensus sequences (CCS). CCS reads with more than 5 passes were selected to minimize the chance of having random errors left in CCS reads.

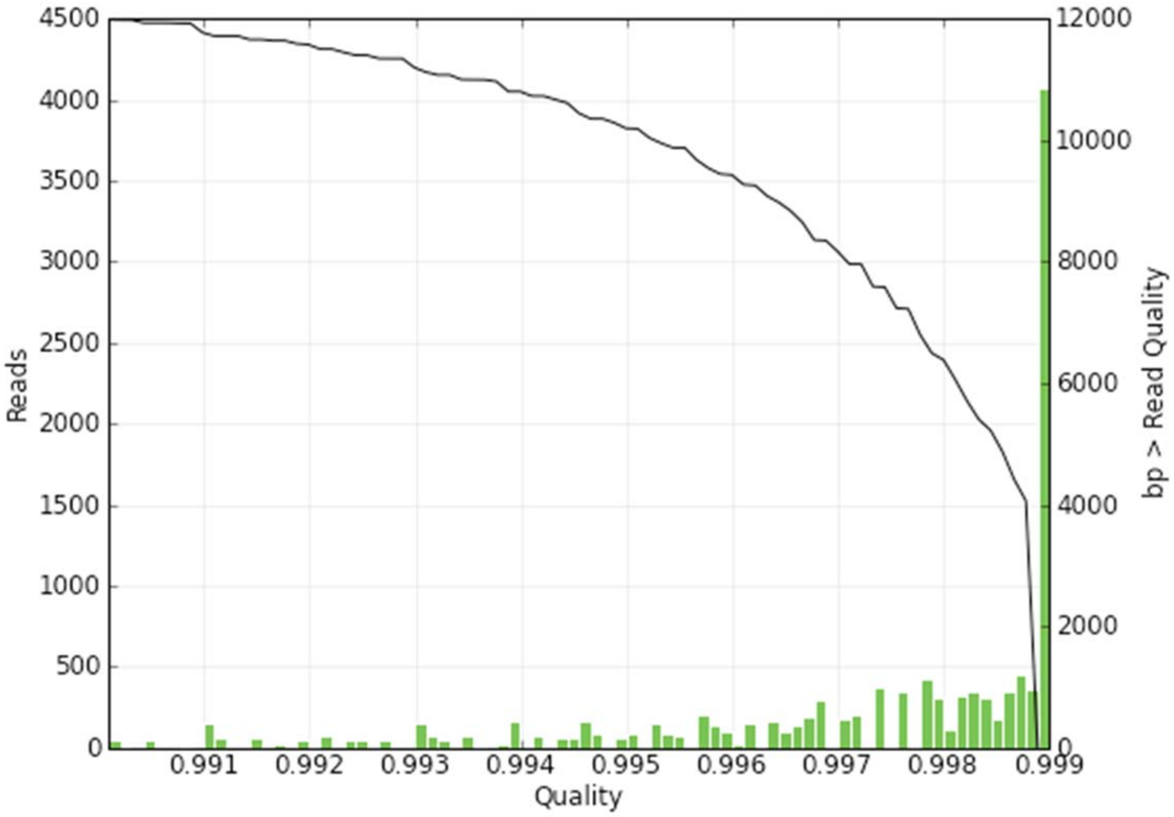

Supplemental figure 3. Quality distribution of circular-consensus reads of ZIKV SL1602 single molecule sequencing. Reads with less than 0.99 quality were removed to allow for reliable detection of minor variants. The black line is the cumulative curve for the number of bases.
